# Development of a Three-Finger Adaptive Robotic Gripper to Assist Activities of Daily Living

**DOI:** 10.3390/designs8020035

**Published:** 2024

**Authors:** Mahbubur Rahman, Tanzil Shahria, Samiul Haque Sunny, Mahafuzur Rahaman Khan, Emroze Islam, Asif Al Zubayer Swapnil, David Bedolla-Martínez, Mohammad H Rahman

**Affiliations:** 1 Mechanical Engineering, University of Wisconsin-Milwaukee, Milwaukee, WI 53211, USA; 2 Industrial Engineering and Management, Khulna University of Engineering & Technology, Khulna 9203, Bangladesh; 3 Computer Science, University of Wisconsin-Milwaukee, Milwaukee, WI 53211, USA; 4 Instituto de Ingeniería Industrial y Automotriz, Universidad Tecnológica de la Mixteca, Huajuapan de León, Oaxaca 69000 Mexico

**Keywords:** robotic hands, assistive grippers, ADLs

## Abstract

A significant number of individuals in the United States use assistive devices to enhance their mobility, and a considerable portion of those who depend on such aids require assistance from another individual in performing daily living activities. The introduction of robotic grippers has emerged as a transformative intervention, significantly contributing to the cultivation of independence. However, there are few grippers in the fields, which help with mimicking human hand-like movements (mostly grasping and pinching, with adoptive force control) to grasp and carry objects. Additionally, the data are not available even on how many Activities of Daily Living (ADL) objects they can handle. The goal of the research is to offer a new three-fingered gripper for daily living assistance, which can both grasp and pinch with adaptive force, enabling the capabilities of handling wide-ranging ADL objects with a minimal footprint. It is designed to handle 90 selective essential ADL objects of different shapes (cylindrical, irregular, rectangular, and round), sizes, weights, and textures (smooth, rough, bumpy, and rubbery). The gripper boasts a meticulously engineered yet simple design, facilitating seamless manufacturing through 3D printing technology without compromising its operational efficacy. The gripper extends its functionality beyond conventional grasping, featuring the capability to pinch (such as holding a credit card) and securely hold lightweight objects. Moreover, the gripper is adaptable to grasping various objects with different shapes and weights with controlled forces. In evaluation, the developed gripper went through rigorous load tests and usability tests. The results demonstrated that the users picked and placed 75 objects out of 90 daily objects. The gripper held and manipulated objects with dimensions from 25 mm to 80 mm and up to 2.9 kg. For heavy-weight objects (like books) where the centroid is far apart from the grasping areas, it is difficult to hold them due to high torque. However, objects’ textures have no significant effect on grasping performance. Users perceived the simplicity of the gripper. Further investigation is required to assess the utility and longevity of grippers. This study contributes to developing assistive robots designed to enhance object manipulation, thereby improving individuals’ independence and overall quality of life.

## 1. Introduction

Over the last decades, the prevalence of upper limb dysfunctions (ULDs) has escalated at an alarming rate, primarily due to stroke, spinal cord injury (SCI), cerebral palsy (CP), multiple sclerosis (MS), amyotrophic lateral sclerosis (ALS), trauma, occupational injuries, and geriatric disorders [1,2]. Stroke is identified as the leading cause of ULDs, accounting for 34% of cases, followed by SCI (27%), MS (19%), and CP (8%) [3]. This condition severely limits an individual’s independence, impacting employment, domestic life, and social participation and often relies on wheelchairs and assistive devices. Over 6.8 million Americans use assistive devices to help them with mobility, 1.7 million are wheelchair users, and almost one-third of mobility device users need assistance from another person in one or more of the ADLs [4]. Notably, an average annual increase of 5.9% in wheelchair usage has been observed [5]. Wheelchair users often report significant limitations in both basic ADLs (BADLs) and instrumental ADLs (IADLs) [6]. The inability to perform essential ADLs can lead to unsafe living conditions and a diminished quality of life [7]. For individuals with a ULD, the capacity to perform self-care without significant assistance is crucial. Current solutions, such as caregivers and assistive devices like reachers and adaptive utensils, support specific tasks. Therefore, there is an immense need for research on ADL assistance targeting people with ULD to enhance their independence and reduce the caregiving burden on families. These ADLs encompass a wide range of occupations, spanning from handling and transporting objects to performing complex movements that replicate the dexterity exhibited in human hands [8]. Numerous researchers have actively engaged in advancing this academic field.

The development of assistive robotics marks a significant advancement in aiding individuals with disabilities, particularly in enhancing their ability to perform ADLs [9]. This need is highlighted by the substantial number of disabled individuals in the United States who require assistance with ADLs, impacting not only their independence but also placing a considerable burden on caregivers and healthcare systems [10]. Robotic grippers, designed to emulate human hand movements, play a crucial role in this context. These devices enable the grasping and manipulating of various objects, facilitating routine tasks that might be challenging or impossible for those with physical limitations. Integrating such technology into daily life is key to enhancing user autonomy and quality of life. The landscape of robotic grippers has evolved significantly, with various designs emerging to address the diverse needs of users, particularly those with disabilities. Rigid links grippers [11], such as those using lead screws [12], offer energy efficiency through self-locking mechanisms but are hindered by slow motion [13]. Handling fragile objects remains a challenge, leading to the development of softer grippers in soft robotics [14]. Innovations like gecko-inspired grippers [15] and soft robotic grippers [16] demonstrate adaptability to different object shapes but face bulkiness and carrying capacity limitations. Traditional grippers with rigid fingers, while effective for simple tasks, fall short in complex manipulations [14,17], and underactuated grippers [18] offer versatility but may lack the force needed for certain applications [19,20]. These limitations underscore the need for a more adaptable, efficient, and versatile gripper design capable of addressing the nuanced requirements of individuals with disabilities in performing daily living activities. Despite notable advancements in robotics, particularly in the area of gripper creation for assisting individuals in diverse occupations, the current solutions remain lacking in terms of comprehensiveness. The current market lacks multifunctional grippers capable of emulating the human hand’s dexterity over a wide spectrum of ADL objects. This scarcity arises due to the prevailing trend of gripper designs that cater to specialized functionalities rather than offering versatility.

Hwang et al. [21] engineered a high-payload soft gripper using electroadhesion, featuring a mechanically reinforced structure. This industrial gripper, equipped with two flexible polyimide film fingers and a multilayered dielectric elastomer actuator, demonstrated versatility in gripping cylindrical bottles, spherical light bulbs, hexahedral polyethylene terephthalate (PET) boxes, flat paper boxes, chocolate packed with PE film, and concave shaped PET bottles. The gripper can effortlessly lift and transport objects of diverse shapes that are 100 times heavier than the gripper itself, which weighs 6.2 g. However, it has yet to undergo testing for handling everyday objects in a home environment. Moreover, the two-finger gripper generally suffices for most applications; the requirement for enhanced accuracy and precision in handling delicate and fragile items justifies the utilization of the three-finger gripper type [22].

Yoon et al. [23] conducted an analysis of the fingertip force vector concerning two-finger grippers (SARAH) designed for robust environmental adaptation. The proposed finger mechanism employs an underactuated four-bar linkage, mirroring the natural movement of the human hand [23]. However, this design choice comes with inherent trade-offs, compromising precision and dexterity [24]. Additionally, akin to the limitations associated with other two-finger grippers [21], this gripper exhibits similar constraints.

Kim et al. [25] introduced the gripper named BLT, an adaptive three-finger gripper with the ability to actively transition between precise pinch and compliant grasping modes. Despite employing a simplified algorithm, the gripper exhibits ample capability for various grasping tasks and achieves a high payload capacity. However, it is noteworthy that the gripper has undergone experiments involving objects of different shapes, providing limited insight into its performance concerning texture, size, and weight, especially in the context of ADL objects.

Hernandez [26] devised a three-finger gripper tailored for assistive robots (GARs) to aid in ADLs. The gripper’s actuators, outfitted with worm gear mechanisms, possess force-sensing capabilities solely during pushing actions, neglecting static force measurement. However, object manipulation often induces reorientation, altering the forces exerted on grasped objects, necessitating static force sensing for intricate shapes [27]. Moreover, while the GAR effectively grasps objects, it lacks the ability to pinch, which is essential for securing lightweight items like credit cards. As a consequence, it diminishes the capacity to manipulate numerous ADL objects [28].

This undertaking is motivated by the acknowledgment of a substantial gap in the functionality of prevailing gripper designs, notably their effectiveness in handling a diverse array of ADL objects, primarily due to the absence of (a) simultaneous pinching and grasping capabilities and (b) adaptive static force application. The principal aim is to address this deficiency in the realm of assistive robotics through the meticulous design and development of an innovative three-finger gripper, engineered to adeptly grasp and pinch objects with adaptable force. The design is grounded in minimalism, resulting in an efficient and cost-effective product that can be easily manufactured using 3D printing technology. This approach not only ensures economic viability but also promotes accessibility. The gripper’s adaptability is a cornerstone of its design, enabling it to proficiently grasp objects of varying shapes and weights with precise force regulation. This feature is enhanced by integrating a customized linear force feedback system, allowing for the adjustment of grip force in accordance with the object’s characteristics, thereby ensuring the safe handling of both hard and soft objects. Additionally, the gripper’s design allows it to fully fold when closed, a feature that optimizes space utilization and adds to its practicality.

The study’s contribution extends beyond mere functionality; it represents a significant stride in the evolution of assistive devices, aligning technological advancement with the nuanced needs of those it aims to assist. A comprehensive series of usability and load tests were conducted to evaluate the feasibility and effectiveness of the newly developed three-finger robotic gripper. The results of these tests are illuminating, offering valuable insights into the practical application of the gripper in assisting with ADLs. The gripper exhibited remarkable adaptability, handling objects varying in size up to 80 mm and managing weights up to 2.9 kg, and while the results are promising, they represent an initial step in developing assistive robots for ADLs. Further research is necessary to evaluate these grippers’ enhanced functionality and durability in everyday tasks. This study’s findings demonstrate the gripper’s potential in practical applications and lay the groundwork for future advancements in assistive robotics.

The rest of the manuscript is structured to present the research and its findings methodically. Section 2 delves into the design and development process of the advanced robotic gripper, highlighting the design objectives, prototype creation, and the integration of the gripper with a robotic arm for testing in ADLs. Section 3 details the experimental setup and procedures, including the selection of materials and components, the design of the gripper’s fingers, and the electronic control system. Section 4 offers a comprehensive analysis of the usability testing and load tests, showcasing the gripper’s efficacy in handling various objects and user feedback on its simplicity and effectiveness. Section 5 explores the implications of these findings, particularly the impact of the three-finger adaptable robotic gripper in assistive robotics and its potential to enhance the independence and quality of life for people with disabilities. Conclusion in Section 6 encapsulates the study’s contributions to assistive robotics, emphasizing the innovative aspects of the gripper and its significance in improving the autonomy and well-being of individuals reliant on assistive technology for ADLs.

## 2. Methodology

This research embarked on the development of a sophisticated 3-finger adaptive robotic gripper, focusing on advancing its intelligent functionalities to support activities of daily living (ADLs). The initial phase entailed delineating clear design objectives that encapsulated the essential capabilities required for effectively assisting individuals with ADLs. Following the establishment of these objectives, the methodology progressed to the conceptualization and computer-aided design (CAD) of the prototype. The manufacturing process leveraged 3D printing technology for rapid and cost-effective prototype development. Subsequent iterations refined the prototype to enhance its durability and reliability in anticipation of extensive testing. The culmination of this development process saw the integration of the gripper with a robotic arm, facilitating its comprehensive evaluation in performing ADLs within a controlled laboratory environment mimicking home scenarios at the UWM-BioRobotics Lab. This methodological approach underscores the commitment to creating a versatile and user-friendly assistive device, tailored to meet the diverse needs of individuals requiring support with daily tasks.

### 2.1. Design Objectives

The design parameters were established to construct a minimum viable solution for a functional robotic gripper covering a maximum amount of ADL tasks and carrying objects up to 2.5 kg weights in horizontal mode and 1 kg weights in vertical mode. The constraints encompass the restriction of three fingers, where each finger is limited to having only one active degree of freedom (DoF) and one passive degree of freedom [26]. This facilitates it to grasp the parallel gripping mode and cylindrical gripping mode and partial spherical mode. Plastic components have been selected for the gripper to make the manufacturing cost-effective. Further, design, development, and evaluation became fast due to the 3D printing components.

### 2.2. Materials and Components Selections

This section comprises the choices of materials and other components selected for the assembly of the gripper.

#### 2.2.1. Materials

The gripper is equipped with a material that demonstrates the ability to securely grasp objects of weight up to 2.5 kg. The mechanical components employed in this system are fabricated using polylactic acid (PLA) [29] via the technique of additive manufacturing, specifically 3D printing. PLA, a thermoplastic polymer, demonstrates superior printability and strength when compared to acrylonitrile butadiene styrene (ABS), thermoplastic polyurethane (TPU), and nylon materials [30]. The utilization of 3D printing technology assisted the expeditious conversion of a computer-aided design (CAD) model into a functional prototype [31]. The integration of various components was accomplished by using adhesives, M3 wood screws, and M2 nuts and bolts.

#### 2.2.2. Actuators

To drive the motion of each finger, it utilized a set of coreless DC motors, the MightyZap 12Lf-100PT-27 (Parameters are shown in Table 1). Each actuator is equipped with an integrated lead screw mechanism, enabling the conversion of rotational motion into linear actuation, thereby allowing for a maximum applied force of 100 N at a duty rate below 20% [32]. Additionally, an incorporated potentiometer (absolute position sensor) is embedded within the motor to ensure precise and high-resolution monitoring of the spring’s position force realtime force feedback [33].

#### 2.2.3. Electronic Control System and Motor Driver

A customized circuit board was specifically developed to streamline the integration of the control components for the robotic gripper. This board features an Arduino Nano housing an ATmega328 microcontroller [34]. Furthermore, the circuit board includes three compact DC-DC 4R7 converters, which efficiently step down the power supply voltage from 24 V to 12 V [35]. This configuration enables precise position control, enabling the gripper to perform actions such as opening, closing, or pinching. Moreover, each finger is equipped with a tailor-made force sensor, providing continuous force feedback that can be adjusted for different grasping forces depending on the specific object being handled.

### 2.3. Mechanical and Electrical Design

This portion covers the design of the fingers and their spatial orientations, followed by an explanation of the organization and control of electronic content.

#### 2.3.1. Finger Design

**Figure 1.**
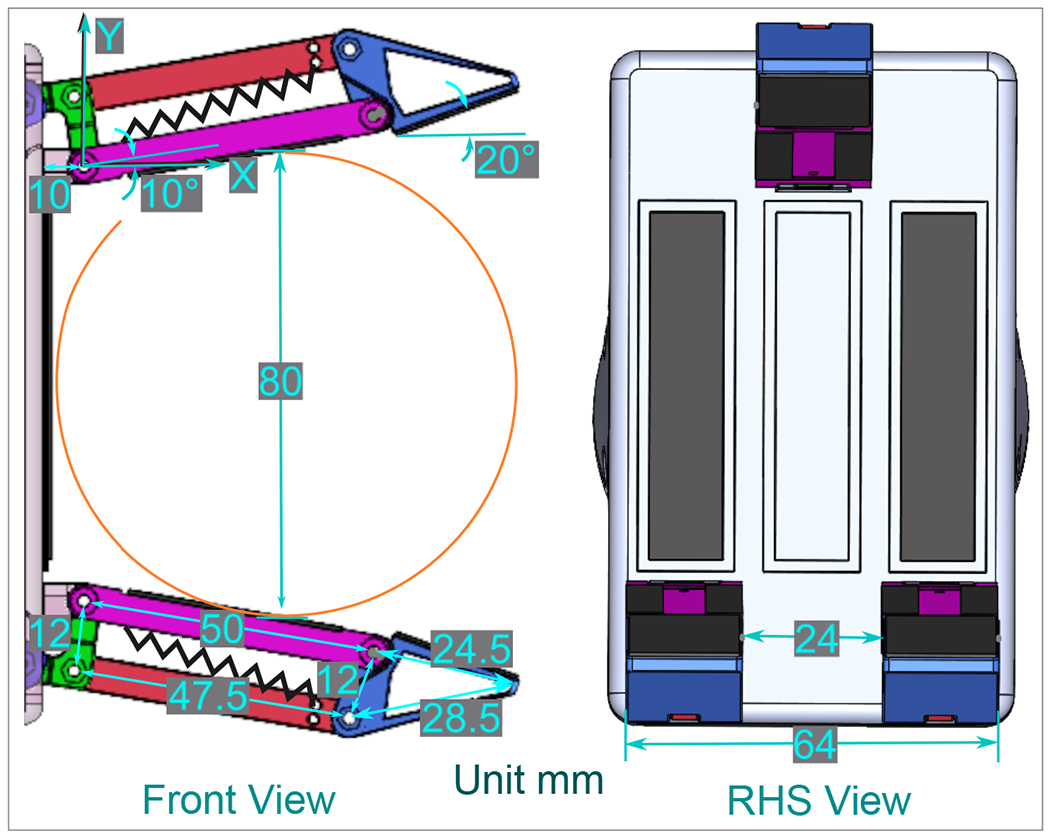
Spatial position of the flanges of the gripper.

**Figure 2.**
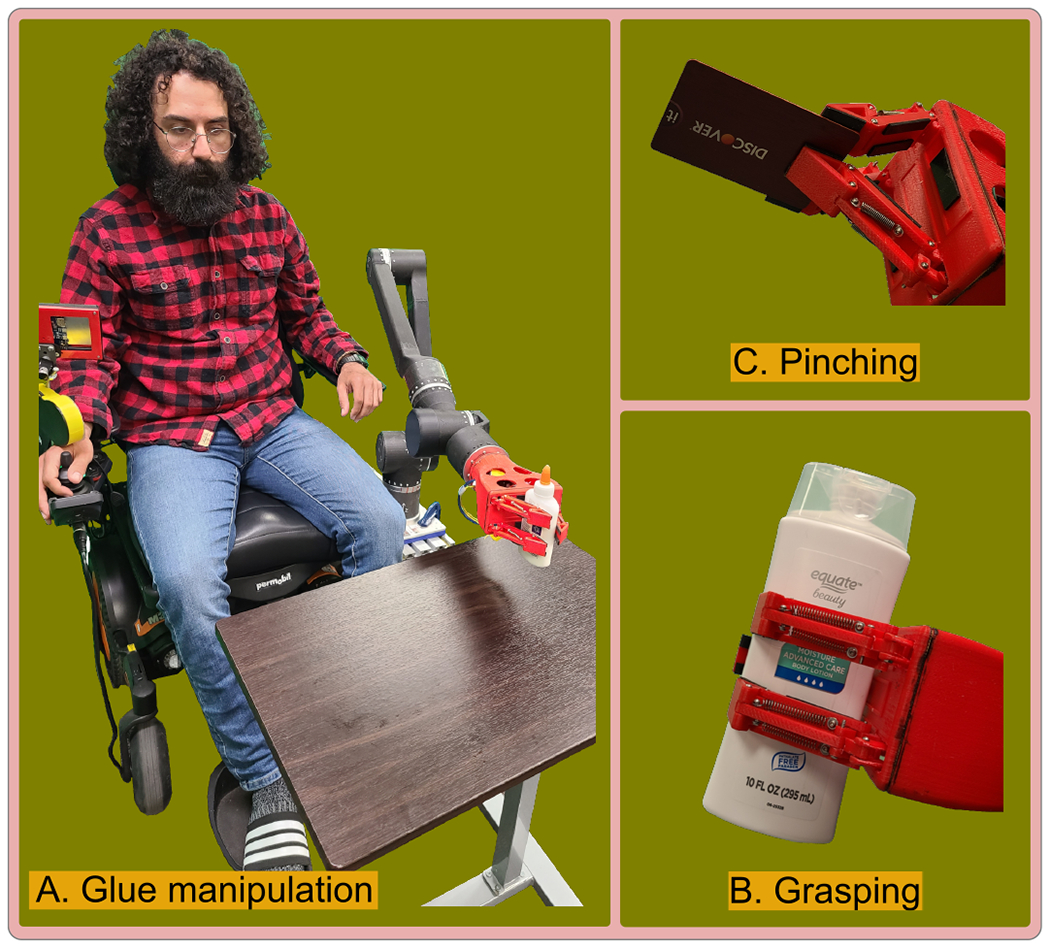
The figure captures the gripper performing three distinct functions: (**A**) Glue manipulation, showing the gripper’s fine motor control and dexterity. (**B**) Grasping, illustrating the gripper’s ability to hold larger objects securely. (**C**) Pinching capability, where the gripper adeptly handles slim objects like a credit card, showcasing the precision and versatility.

In [36], the authors introduce an optimized gripper with a geometric design tailored for a three-phalanx underactuated finger. The primary objective is to enable the gripper to execute three distinct types of grips: spherical, parallel, and cylindrical. As highlighted in [37], human grasping typically involves three fingers—the index, middle, and thumb—prompting the selection of three fingers in the design to align with this natural functionality. Additionally, to enhance simplicity, the design adopts a two-phalanx mechanism, mirroring the approach outlined in the study [36]. In this proposed gripper, each underactuated finger mechanism possesses two degrees of freedom. The incorporation of a passive element, specifically a spring positioned between the first and second finger phalanges, activates the second degree of freedom. Figure 1 illustrates the schematic design of the finger, featuring two phalanges, two links, a spring, and a third-grade connector element that transforms linear motor motion into rotary motion for the mechanism. Spring’s presence allows the finger to rotate around a fixed pivot, resembling a single rigid body. When the first phalanx contacts a surface, the actuator’s force stretches the spring, transmitting motion exclusively to the second phalanx [36]. The gripping motion concludes when the two phalanges come together to grasp an object, completing the full grasp, as depicted in the comprehensive design presented in Figure 2. This design showcases all the key elements within the system, aligning with the gripper’s multifunctional and adaptable grip capabilities.

#### 2.3.2. Spatial Position of the Flanges

The configuration of the palm holds significance, influencing the angles between the fingers and their coordination during the act of grasping. The spatial alignment of these three fingers is crucial to create a gripper with the capability to securely hold objects within a diameter range of up to 80 mm [26]. The test objects used in these assessments serve as benchmarks, representing the minimum and maximum specifications for both length and diameter relevant to each type of grip. Figure 1 shows the spatial position of the fingers, where the illustrated arrangement of the components facilitates holding cylindrical objects of a maximum diameter of 80 mm. Besides holding, the side where two fingers are located prevents torque generated by the objects, rendering a firm grip.

**Figure 3.**
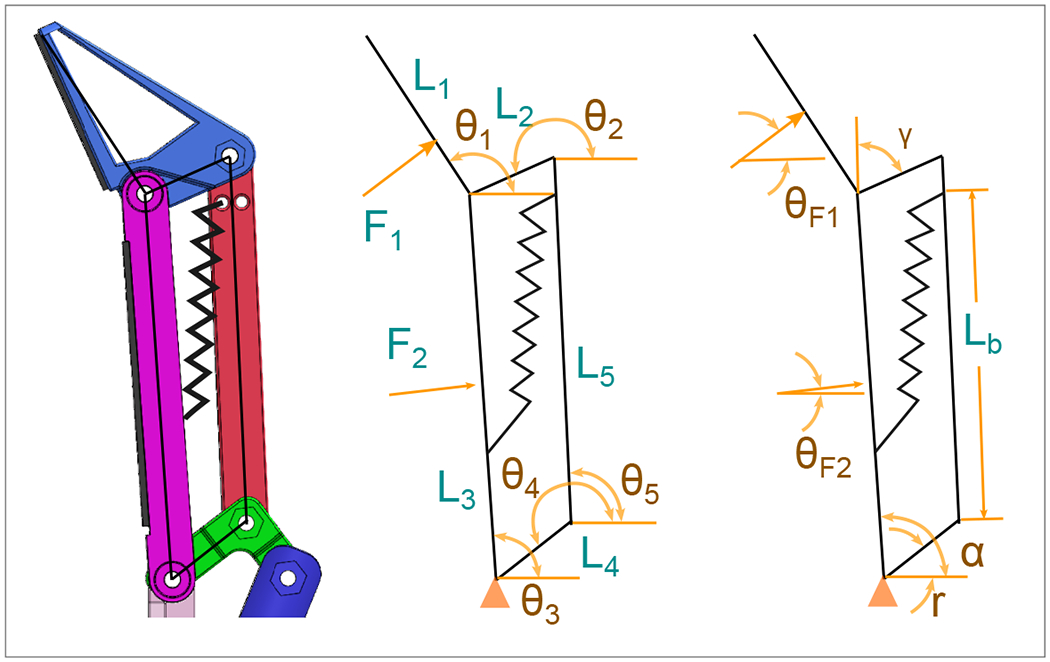
Vectorial representation of the finger [26].

The utilization of kinematic and dynamic analyses is fundamental in understanding the motion and behavior of mechanical systems, particularly those characterized by underactuation. This study employs principles of kinematics and dynamics to analyze an underactuated gripper mechanism (Figure 3), as previously designed by Hernandez [26].

Here, the positions of each phalanges,

(1) $$\theta_{1}=90^{\circ }+\theta_{F1}$$

(2) $$\theta_{2}=180^{\circ }-r$$

(3) $$\theta_{3}=90^{\circ }+\theta_{F2}$$

(4) $$\theta_{4}=180^{\circ }-\gamma$$

(5) $$\theta_{5}=\tan^{-1}\left(\frac{L_{3}Cos\theta_{3}-L_{2}Cos\theta_{4}-L_{4}Cos\gamma }{L_{3}Sin\theta_{3}-L_{2}Sin\theta_{2}-L_{4}Sin\gamma }\right)$$

Linear force of the motor,

(6) $$F_{\text{Motor}}=L_{4}\left(\frac{-\left(L_{5}-L_{b}\right)\cos(\alpha )F_{k}}{L_{5}L_{6}}\right)$$

#### 2.3.3. Design of Phalanges for Pinching

**Figure 4.**
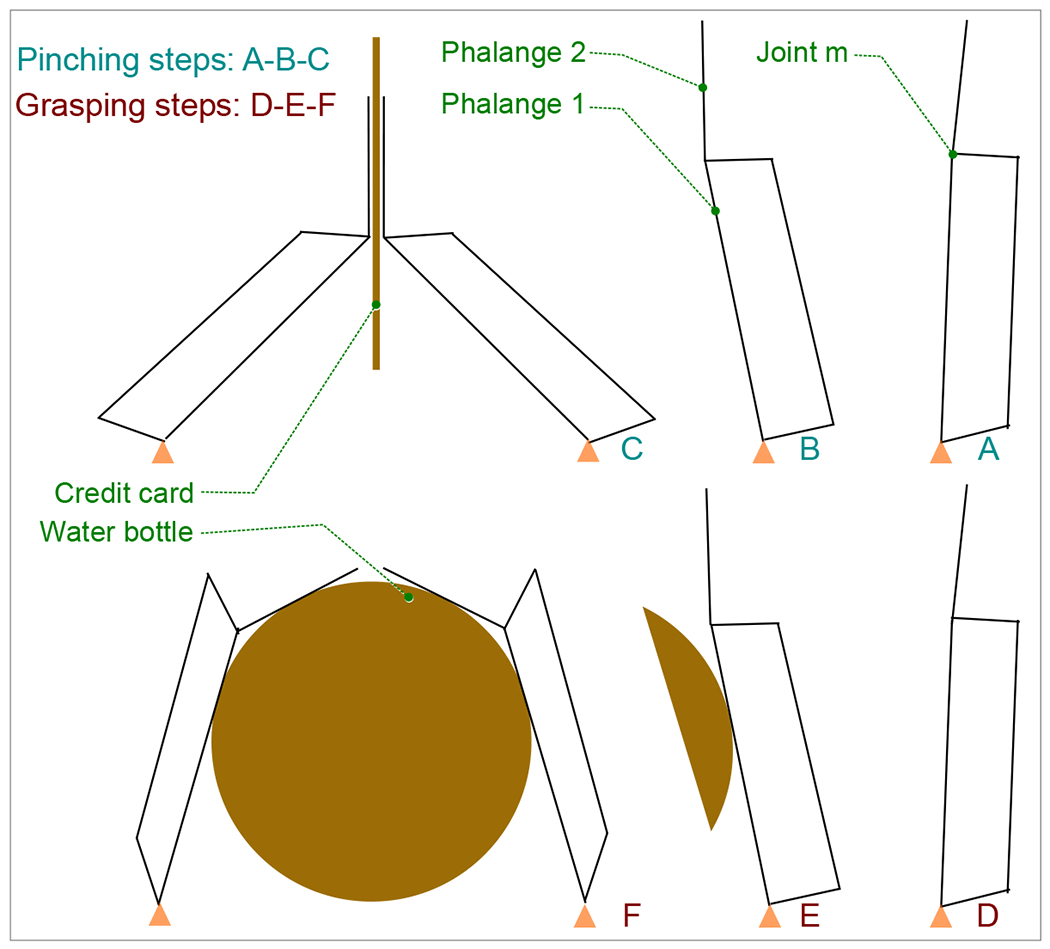
Steps in pinching (credit card) and grasping (water bottle).

This design integration of pinching functionality alongside traditional grasping capabilities enhances the versatility and adaptability of the gripper, making it suitable for a broader range of manipulation tasks in various applications. In the proposed mechanism, illustrated in Figure 4. The design of phalanges for pinching in the gripper mechanism involves careful consideration of the lengths of the phalanges and the limit of the joint motion (joint m). These parameters are crucial for enabling the gripper to perform both pinching and grasping actions effectively.

The gripper is capable of executing pinching motions in addition to conventional grasping maneuvers. The design is optimized to facilitate pinching when external forces are not exerted on phalange 1, as depicted in steps A-B-C of the mechanism. During this operation, phalange 2 of the opposing finger remains parallel to effectively secure objects such as credit cards. Conversely, during grasping actions (illustrated in steps D-E-F), external forces are applied to phalange 1 initially. Subsequently, phalange 2 rotates to exert pressure on the object, enhancing the grip strength and stability of the gripper.

#### 2.3.4. Force Sensor Design

**Figure 5.**
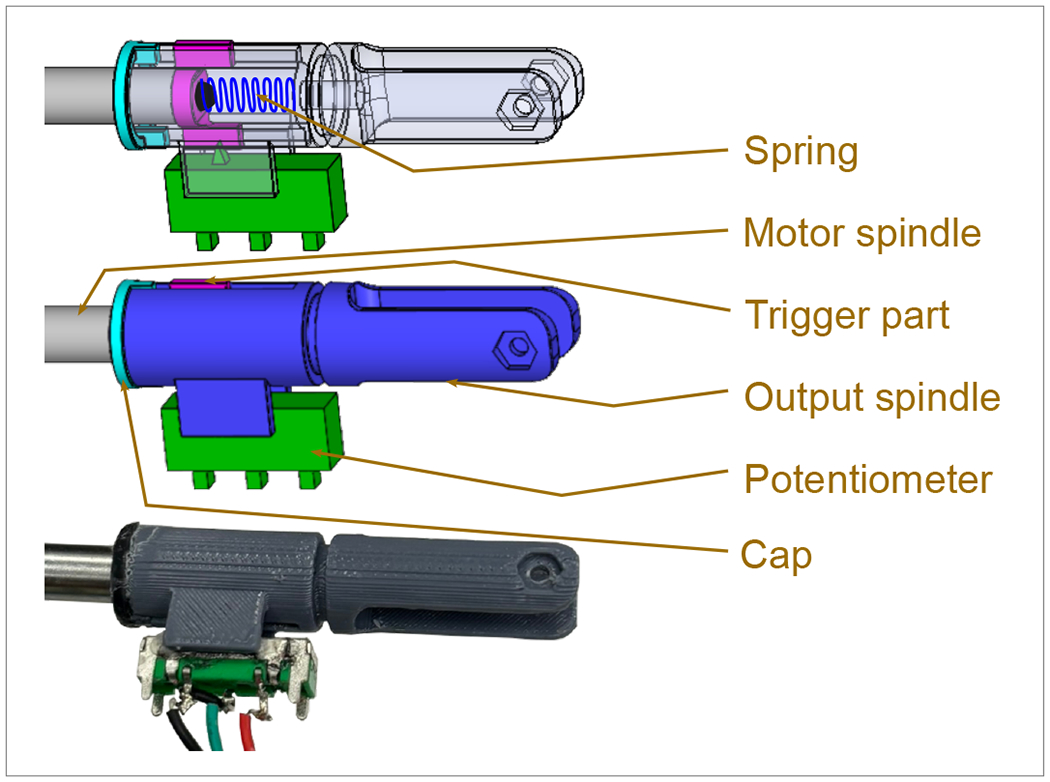
Customized force sensor for adaptable grasping.

The proposed gripper incorporates our developed force sensors (depicted in Figure 5). The primary component is a linear potentiometer, which undergoes adjustment by the trigger part whenever any relative movement occurs between the motor spindle and the output spindle. This relative movement transpires when there is a change in the applied force on the spring. The controller is equipped with calibration for force sensing based on the potentiometer position. It continuously reads the potentiometer position, representing the force, at one-millisecond intervals, allowing it to make real-time decisions regarding the finger’s position. As a result, the controller can apply appropriate pressures to objects based on their shape, size, and weight, rendering the gripper adaptable for handling items ranging from rigid to delicate parts.

#### 2.3.5. Circuit Board Design

**Figure 6.**
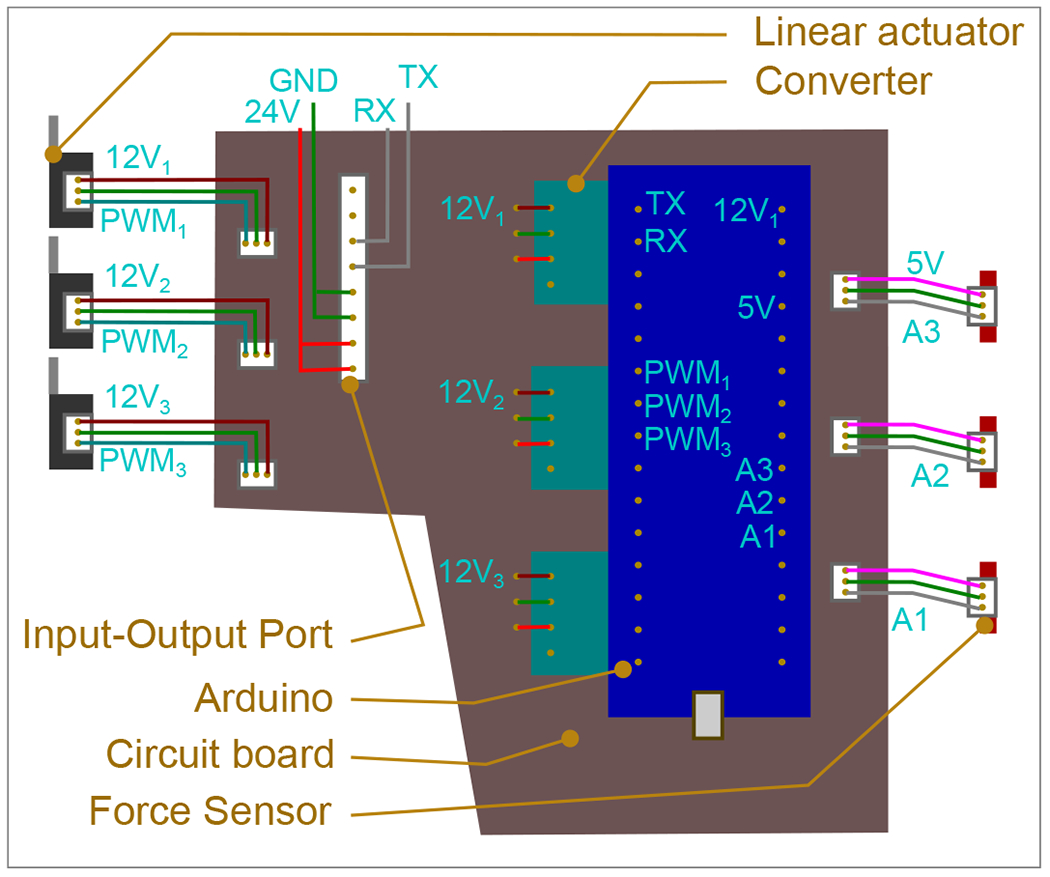
Overview of the circuit board components, detailing the color-coded wiring scheme. Bright red denotes the 24 V supply, dark red signifies the 12 V supply, pink represents the 5 V supply, green denotes ground connections, gray indicates signal lines, and cyan represents PWM connections.

**Figure 7.**
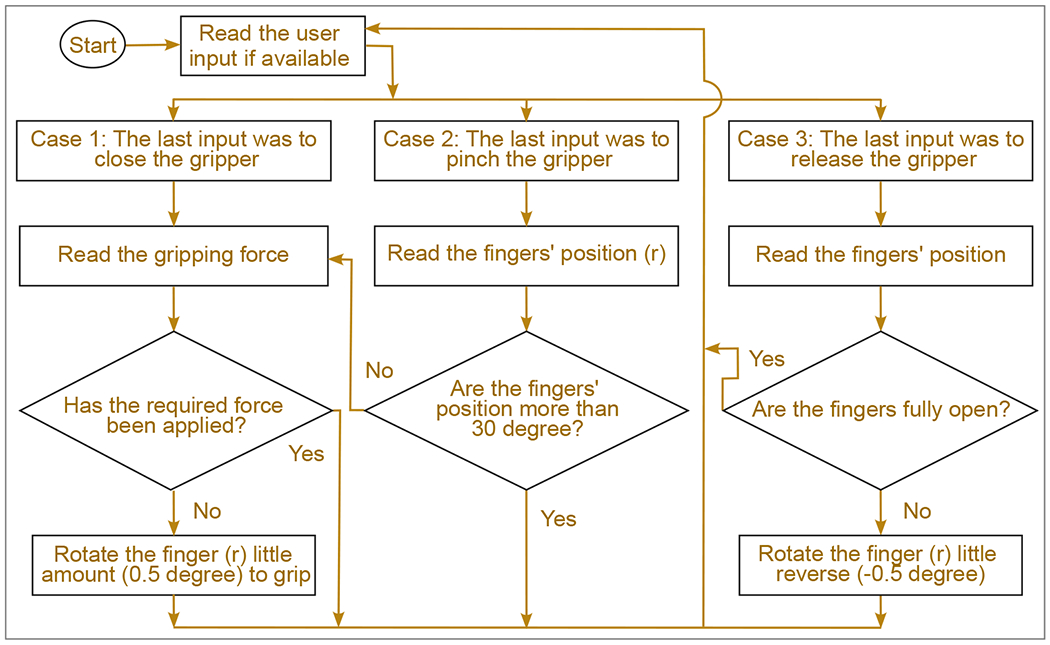
Control algorithm of the gripper.

**Figure 8.**
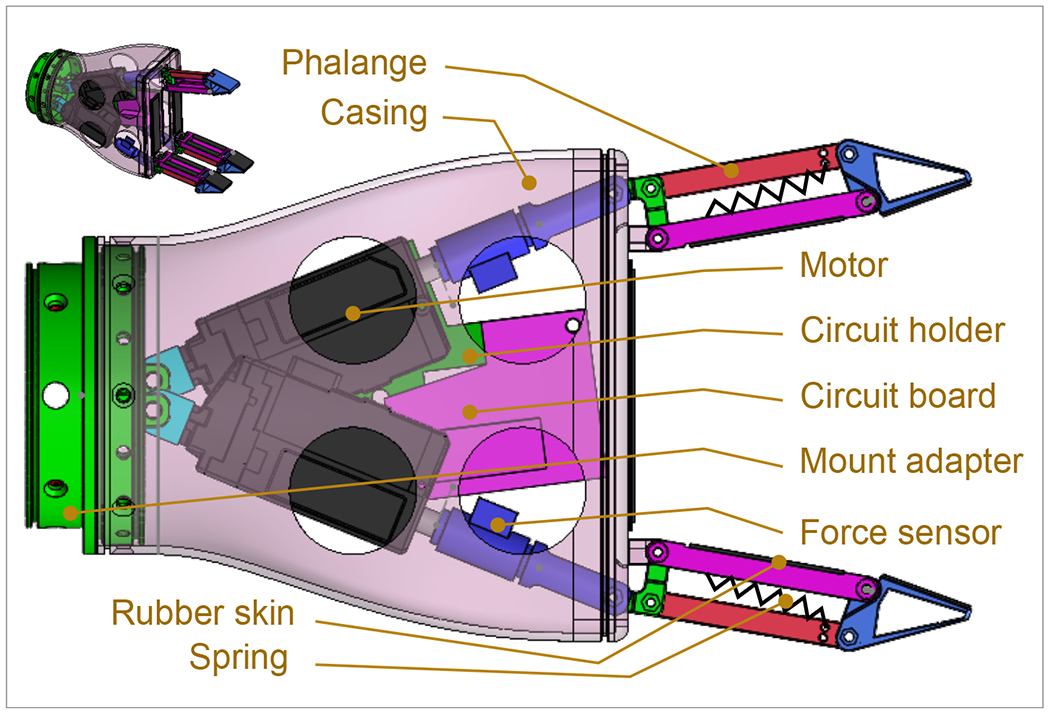
The CAD model of the gripper with internal mechanism.

The circuit (Figure 6) receives a 24 V power supply, and this voltage is parallelly converted to 12 V by three compact buck converters for the three motors. One of these converters also provides power to the Arduino Nano, while the force sensor receives a 5 V supply from the Arduino. Acting as a controller, it sends 5 V PWM Digital Logic inputs for motors. On the other hand, it sends and receives commands by serial communication with the robotic arms [39]. The Arduino reads user commands and monitors the force applied by the three motors at one-millisecond intervals. Based on this real-time data, the Arduino determines the precise positioning and actuation levels required for each motor. Figure 7 shows the control algorithm of gripping, pinching, and opening actions. All the electrical components are assembled and hung to an actuator shown in Figure 8.

### 2.4. Manufacturing and Prototyping

**Figure 9.**
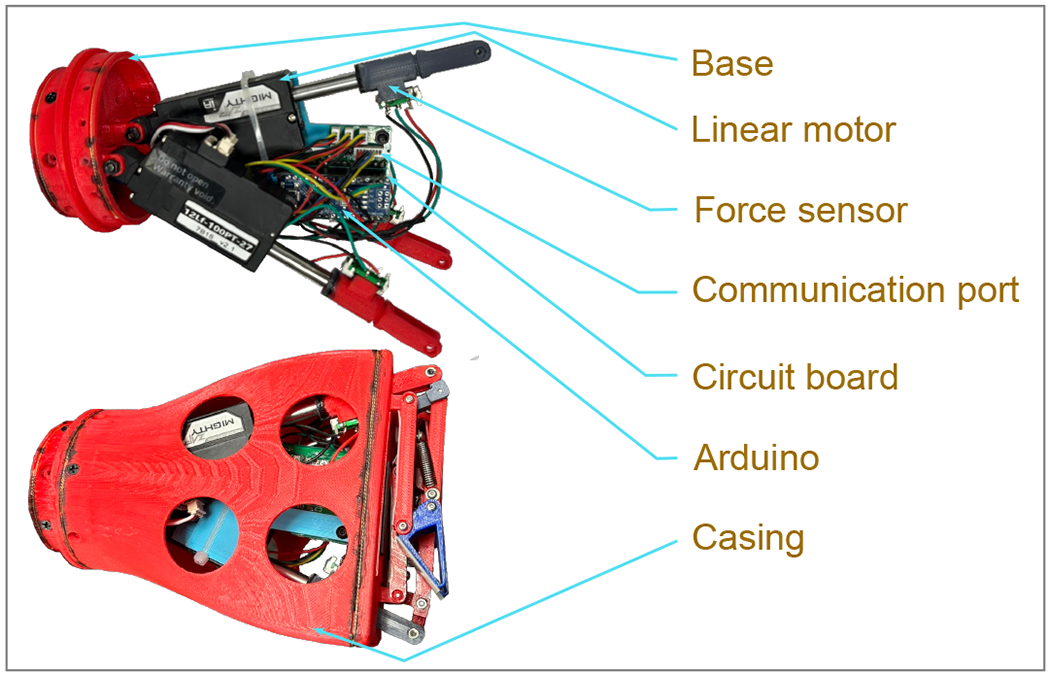
The 3D-printed prototype of the developed gripper.

The fabrication process was fused deposition modeling (FDM) 3D printing techniques with PLA filaments (Figure 9). The key actuators, located at Joint-1, consist of coreless DC motors of the MightyZap 12Lf-100PT-27 model [38]. The actuators are connected to the base with M3 bolts, facilitating revolute joints. With the same bolt, the force sensors are attached to the spindle of the actuators. Then, the base-actuators–sensors assembly is inserted into the case, where 8 M3 wood screws hold the base and case together. After that, the fingers are attached to the other side of the force sensors. In fingers, phalanges are connected by M2 bolts, where every joint provides rotating motions. Finally, the gripper was mounted to the wheelchair-mounted multifunctional robotic assistive arm (mR2A) (Figure 2), developed in BioRobotics Laboratory, University of Wisconsin Milwaukee. This comprehensive integration of electronic control and fabrication techniques forms a robust foundation for the efficient functioning of the robotic gripper within the UWM-BioRobotics Lab’s environment.

## 3. Experimental Study

This study investigates the designed three-finger adaptive robotic gripper, which is purposed to augment daily living activities. This segment articulates the systematic framework instituted for the evaluation, encompassing participant selection criteria, the construction of the experimental milieu, the protocols for data acquisition, and the criteria for assessing the gripper’s efficacy.

### 3.1. Study Site and Participants

The experimental work was conducted at the BioRobotics Laboratory, University of Wisconsin–Milwaukee. A total of six individuals who were in good health and had an average age of 26.3 years with a standard deviation of 2.3 years were included as participants in this research investigation.

### 3.2. Experimental Setup

**Figure 10.**
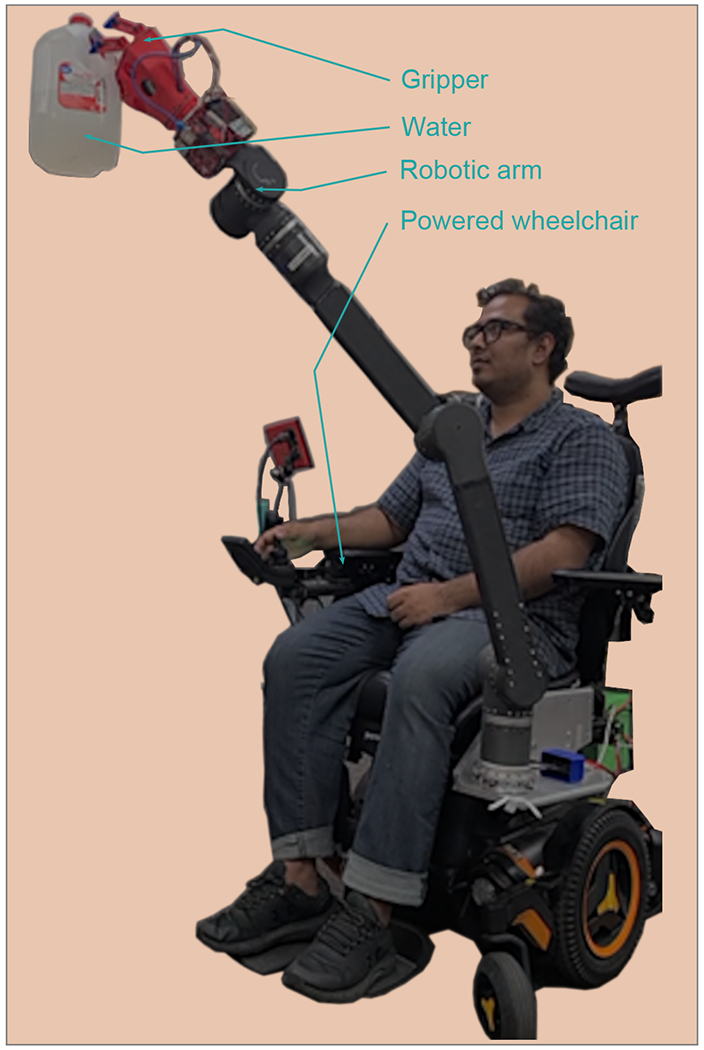
Load testing with mR2A robot.

**Figure 11.**
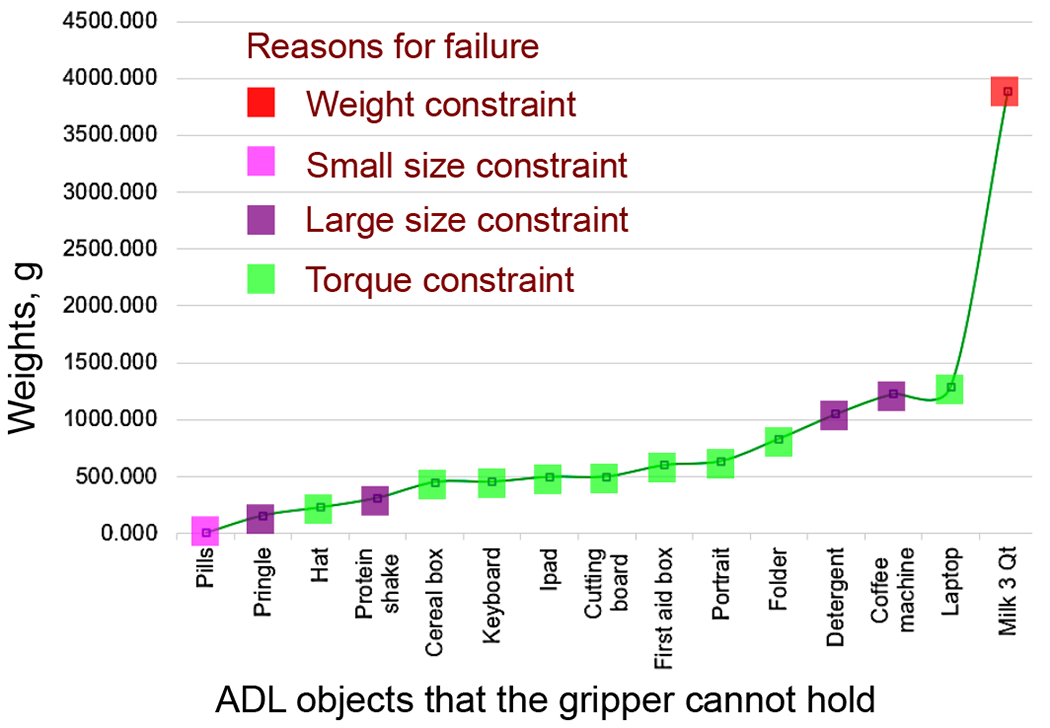
Analyzing the failure reasons of the gripper for some ADL objects.

The experimental setup mirrored real-life conditions (Figures 10 and 11), featuring an assortment of 90 distinct ADL objects (see Table 2) chosen for their commonality in household settings and their varied shapes, sizes, textures, and weights [40]. These objects were manipulated using a robotic arm equipped with the gripper, controlled via a joystick interface to reflect the user experience of individuals with limited upper limb movement.

### 3.3. Data Collection Furthermore, Evaluation Criteria

Data collection was comprehensive, capturing both quantitative metrics such as the number of successful grasps, object weights, dimensions, and forces applied and qualitative feedback regarding usability and user satisfaction. We meticulously defined evaluation criteria centered on the gripper’s capability to reliably grasp and manipulate objects without causing slippage, damage, or user strain. Standardized protocols within a controlled laboratory setting were employed to guarantee uniformity across tests, with consistent robotic arm movements and clear participant guidance to reduce variability. The resulting data were subject to thorough statistical analysis, fortifying the validity and dependability of our research outcomes.

### 3.4. Load Test

The experiments involving the three-finger gripper entailed subjecting it to a range of weights until it reached its load-bearing limit (Figure 10). The gripper was affixed to the mR2A robots located within the BioRobotics laboratory at the University of Wisconsin–Milwaukee. The mR2A is a six-degree-of-freedom robotic arm on a wheelchair. The end effector movements of the robot were directed using a joystick. To vary the weight, different quantities of water were poured into a container, ranging from 200 g to 3.2 kg, with increments of 300 g. Following the successful load test of 2900 g, the finger experienced a fracture when subjected to a weight of 3200 g.

### 3.5. Usability Test of the Gripper

The usability test involved a variety of shapes and materials to assess the participants’ interaction with different objects. The shapes were cylindrical, irregular, rectangular, and round, while the materials included delicate fabric, food, and rigid substances. Table 2 presents the object lists categorized based on ADLs and their corresponding weight ranges. This comprehensive range of objects aimed to evaluate the participants’ handling of items with varying shapes, materials, and weights, providing valuable insights into the usability of robotic systems in everyday scenarios.

**Figure 12.**
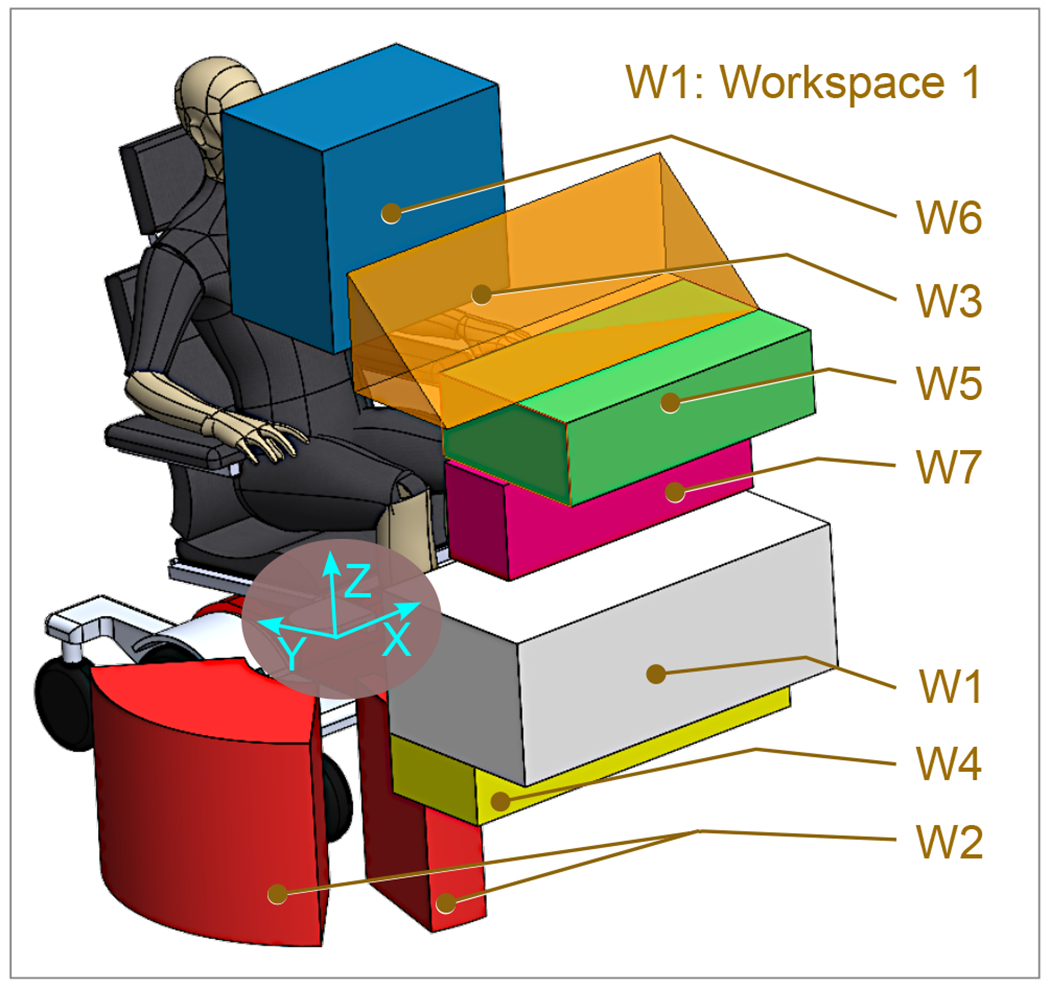
ADL workspace relative to the users and the robot.

The participants utilized an assistive robotic manipulator (Figure 10), which was affixed to a wheelchair, in order to manipulate a variety of objects routinely employed in ADLs in the workspace shown in Figure 12. This was achieved by employing a joystick controlled with a finger. Upon the conclusion of the experiment, a comprehensive usability and satisfaction survey was administered to all participants in order to assess the efficacy of the constructed three-finger-type robotic gripper. First, a two-page instruction sheet was given to the subjects. Then, 90 objects (Table 2) were picked and placed in the ADL workspaces to verify the gripper’s competence. After completing the study, participants were requested to complete a user satisfaction survey.

## 4. Results

The three-finger gripper grasped 75 household items 93% of the time. Compare success rates of grasping, manipulating, and releasing objects of different shapes, sizes, weights, and textures. The gripper can hold 2.9 kg and manipulate objects between 25 mm and 80 mm in at least one dimension. The amount of gripping force exerted is contingent on factors such as the surface texture, weight, and the position of the center of mass of the objects. Objects with greater weight and smoother surfaces necessitate increased gripping force. However, the object’s texture is not important in grasping success because the fingers have silicon skin that facilitates enough contact friction with every object.

On the other hand, the object’s shape is a deciding factor for medium and heavy objects. Items with a center of gravity positioned farther from the gripping point, such as books, require a higher level of gripping force to manage high torque. Consequently, the gripper could not handle books and iPads, though its weight was below the maximum capacity of the gripper (Figure 11).

The gripper demonstrates the capability to execute both grasping and pinching actions with adaptive force (Figure 2), thereby achieving our primary objective. As a secondary aim, the gripper is engineered to fully close during periods of non-operation (Figure 9), thereby minimizing its footprint and safeguarding the fingers from potential damage by eliminating unnecessary protrusions during transportation and storage.

In the conclusive phase of our study, the thorough usability and satisfaction survey unequivocally illustrated the gripper’s user-friendly nature and intuitive control interface. Respondents consistently reported a high level of ease in operating the gripper, validating its efficiency and practicality in ADL applications, thus contributing positively to user satisfaction. This outcome underscores the successful integration of user-centered design principles into the gripper’s functionality, affirming its accessibility and usability across various daily tasks and scenarios.

## 5. Discussion

The developed three-finger adaptable robotic gripper is a significant advancement in assistive robotics, especially considering its potential to improve the independence and quality of life for people needing help with ADLs. The device’s potential is enhanced by its adaptability, simplicity of design, and capacity to manage a diverse array of object sizes and weights. The gripper is capable of pinching and securely holding a wide variety of objects. The usability and load test results demonstrate that the gripper successfully managed to pick and place 75 out of 90 daily objects, showcasing a 93% success rate. This level of performance underscores the gripper’s capability to interact with a broad spectrum of household items, highlighting its adaptability and practical utility in real-world scenarios. The ability of the gripper to handle objects varying in size up to 80 mm and up to 2.9 kg in weight is particularly noteworthy. This range covers a substantial portion of everyday objects, indicating that the design objectives focusing on adaptability and controlled force application were effectively met. A notable aspect of these grippers is their development using 3D printing technology, which underscores the feasibility of short-time, cost-effective production and opens avenues for easy customization and repair.

Furthermore, it is noteworthy to highlight that the gripper’s finger exhibits the capability to achieve closure with a minimal footprint, as illustrated in Figure 9. This particular design feature holds significant implications for enhancing the overall portability of the gripper system. The reduction in spatial requirements for finger closure not only contributes to the compactness of the device but also facilitates ease of transport. Such considerations are pivotal in applications where portability and maneuverability are crucial factors, underscoring the practical and functional advantages of the proposed gripper design. Additionally, the simplicity of the design, as perceived by the users, further adds to the device’s practicality, making it a viable option for individuals who need assistance with ADLs. This simplicity is crucial, as it lowers the barriers to adoption and usage, especially for individuals who are less familiar or comfortable with robotic technology.

Table 3 presents a comparative analysis of benchmarks involving analogous technologies that have evaluations with ADL objects. Alternative grippers have undergone assessments involving 3 to 7 ADL objects or sample objects with four to five distinct shapes. In contrast, the proposed gripper has undergone comprehensive testing with a carefully chosen set of 90 ADL objects, successfully handling 75 of them. This rigorous evaluation underscores the gripper’s effectiveness, particularly in the context of applications related to ADLs.

Even though these results appear beneficial, additional investigation and development are needed in specific segments. The design needs to be improved, and it needs to be tested for more extended periods to see how well it works and how long it endures. The fact that the gripper could not handle 15 of the tested items suggests room for improvement in its design. This might involve enhancing the grip strength, increasing the range of sizes and shapes that can be accommodated, or improving the sensitivity of the gripper to handle more delicate tasks. Additionally, future investigations should focus on long-term performance assessments, including wear and tear under standard usage settings, as well as exploring user experience optimization to ensure that the gripper is not only functional but also easily accessible and enjoyable for all potential users. Moreover, integrating the gripper with various robotic arms and platforms must be explored to enhance its applicability in diverse environments. Compatibility with different robotic systems would increase its utility and make it a more versatile tool for aiding ADLs. Another consideration for future research is that the path forward involves a multidisciplinary approach, combining insights from robotics, ergonomics, user experience design, and direct feedback from end-users to create a more inclusive and efficient assistive tool. While the simplicity of the gripper was well-received, a detailed study on user interaction and control interfaces could provide insights into how to make the device more intuitive and user-friendly, especially for those with limited technical skills or physical capabilities.

Furthermore, the study’s positive findings in terms of usability and accessibility indicate an optimistic future for robots in everyday life. The potential impact on those who require assistance with ADLs is significant, providing a path to better independence and quality of life. This research not only contributes to the field of assistive technology but also paves the way for broader applications of robotics in enhancing daily living, signaling a significant step forward in integrating technology and personal care.

## 6. Conclusions

Our study reveals the impressive performance of the three-finger gripper in grasping a wide range of household items, achieving a success rate of 93%. Through meticulous analysis, we compared the success rates of grasping, manipulating, and releasing objects of different shapes, sizes, weights, and textures. We found that the gripper effectively held objects weighing up to 2.9 kg and manipulated items ranging from 25 mm to 80 mm in at least one dimension. Grip force exertion varied based on factors such as surface texture, weight, and the object’s center of mass position. Notably, the gripper’s silicon skin enabled sufficient contact friction, rendering object texture less significant in grasping success. However, object shape proved crucial for medium and heavy objects, with items like books requiring increased grip force to manage torque.

Despite its remarkable performance, the gripper faced challenges handling certain objects, particularly books and iPads, due to their unique shapes and weight distribution. Nevertheless, our usability survey demonstrated the gripper’s user-friendly design and intuitive control interface, confirming its practicality and efficacy in assisting Activities of Daily Living (ADL). Users consistently reported ease of operation, highlighting the successful integration of user-centered design principles into the gripper’s functionality. Overall, our findings support the gripper’s utility in enhancing independence and quality of life for individuals requiring ADL assistance, marking a significant advancement in assistive robotics.

In future iterations of this research, it is imperative to conduct tests with real subjects to gain deeper insights into the practical usability and ergonomic aspects of the gripper in real-world scenarios. A significant area of focus will be on reducing the size of the gripper to enhance its portability and ease of use while increasing its aesthetic appeal to make it more user-friendly and less obtrusive. The current prototype, constructed using PLA filaments through 3D printing technology, presents an opportunity for material advancement. Transitioning to more robust materials such as aluminum or polyethylene terephthalate glycol (PETG) could substantially improve the durability and longevity of the gripper and increase its payload capacity. However, this material shift necessitates further development and refinement of the current design, particularly in miniaturization, to ensure that the enhanced robustness does not compromise the gripper’s functionality or user accessibility. Integrating more sophisticated sensory feedback mechanisms, such as tactile sensors, could significantly improve the gripper’s ability to adapt to different objects and environments. This enhancement would allow for a more nuanced and sensitive handling of a wider range of objects, particularly those requiring delicate manipulation. Employing machine learning algorithms and artificial intelligence could enable the gripper to learn from its interactions and improve its performance over time. This could involve the gripper adapting its grip based on the object’s weight, texture, and fragility, increasing its efficiency and effectiveness. This evolution is expected to significantly elevate the performance and applicability of the gripper in assisting individuals with daily living activities.

## Figures and Tables

**Table 1.** The proposed gripper at a glance.

Gripper Properties		Actuators, MightyZap 12Lf-100PT-27 [38]	
Materials	PLA	Input voltage	DC 6V-13V
No of Fingers	3	Max Current	1.6 A
DoFs in each finger	2	Stroke	27 mm
No of Actuator	3 (Underactuated)	Linear rated force	100 N
Length of each finger	84.5 mm	Stall force at 1000 mA	48 N
Range of motion (degree) from x-axis	Joint1: −90 to 10, Joint2: −45 to 20	Max speed	7.7 mm/s
Holding capacity	Size: 80 mm, Weight: 2.9 kg	Mechanical self-lock	Yes
Gripping type	Cylindrical, parallel, and partial spherical	Size (Excluding spindle and hinge)	57.4 × 29.9 × 15 mm^3^
Finer position at idle	Fully closed	Weight (Excluding spindle and hinge)	48 g

**Table 2.** Object lists that have been considered in the usability test.

ADL Objects (Italics Are Failed in Grasping)	Weight Ranges
USB memory, Earrings, Tea bags, *Tic tac box* , Necklace, Toilet paper, Ruler, Credit/Debit card, Glue, Tacos, Bookmarker, Glasses, Pair of socks, Bag of chips, Popcorn, bag, Sharpener, Phone charger, *Cereal box*, Toothbrush, Earphones, Pencil, Wallet, Pen, Table lamp	Very light (1 g to 49 g)
Fruit peeler, Scissors, Highlighter, Hand watch, Remote control, Mouse, Deodorant, *Hat*, Lipstick, Avocado, Artificial apple, Marker, Plum, French fries, Eraser, Hard drive, iPod, *Pills*, Slice of pizza, Stapler, *Pringle*, Peach, Plum, French fries, Eraser, Hard drive	Light (50 g to 299 g)
*Protein shake*, Power bank, Shoe, Ketchup, Bag, Knife, Tomatoes can, Umbrella, *Keyboard*, Mouse pad, *iPad, Cutting board*, Moisturizer, *First aid box, Portrait, Folder*, Diary	Medium (300 g to 999 g)
Desk phone, Milk 1 Qt bottle, *Detergent*, Mouthwash, *Coffee machine, Laptop*, Blanket, Book, *Milk 3 Qt*	Heavy (1000 g to 4000 g)

**Table 3.** Bench-markings with similar type of technologies.

Robotic Grippers	Grippers’ Characteristics	Number of Success ADL Objects
Li et al. [15]	Multiple legged; Gecko-inspired adhesion gripper	3 (steel plate, soccer ball, and glass)
Lee et al. [41]	3 fingers; Origami inspired; Flexible and rigid polymer composite; Central servomotor; Cable driven; Underactuated fingers	3 (fabrics, clementine, egg, and rubik’s cube)
BLT Gripper [25]	3 fingers; Fexible belt materials with high-stiffness; ABS structure	3 (driller, pencil, and tape)
Lu et al. [42]	2 fingers; 1 DoF in each finger; Variable friction surface; ABS/TPU material; 2 Dc motors for changing the friction; 2 servo motors for the fingers; Clamp/clip mechanism	7 (custom objects prepared for the experiments)
Li et al. [16]	2 fingers; Soft gripper; It pick and arrange small objects	4 (pliers, tape, haptic device, and screwdriver)
gripper; 2 fingers in each;	5 (screw, washer, tape, pully, and peg)	
Hussain et al. [44]	2 fingers; ABS material; Serrvo motor controlled; Underactuated fingers; Tendon-driven	4 (cup, tennis ball,and small box)
Tlegenov et al. [45]	3 fingers; 3D printed; Thermoplastic elastomer (TPE) /PLA /ABS; Central servo motor; Underactuated fingers; No force feedback	4 (pencil, bottle, witheboard eraser, and ball)
Proposed gripper	3 finger; 3D printed; Underactuated fingers; Force feed back; Customized force sensor; Cylindrical, parallel, and partial spherical; Fingers are fully closed at idle	75 (Table 2)

## Data Availability

The datasets generated during and/or analyzed during the current study are not publicly available due to the conditions of the funding source but are available from the corresponding author on reasonable request.
